# Factors associated with interruption of tuberculosis treatment among patients in Nandi County, Kenya 2015

**DOI:** 10.11604/pamj.supp.2017.28.1.9347

**Published:** 2017-11-06

**Authors:** Alfred Wandeba Wanyonyi, Paul Mutebi Wanjala, Jane Githuku, Elvis Oyugi, Hellen Kutima

**Affiliations:** 1 Jomo Kenyatta University of Agriculture and Technology, Kenya; 2 Field Epidemiology and Laboratory Training Program, Ministry of Health, Kenya; 3 University of Eldoret, Kenya

**Keywords:** Tuberculosis, Treatment interruption, adherence, Kenya

## Abstract

**Introduction:**

Kenya is ranked 15th on the list of 22 high-tuberculosis (TB) burden countries with a case notification rate of 440 cases per 100,000 persons. Interruption of TB treatment is one of the major obstacles to effective TB treatment and control. Since 2009, emphasis has been on direct observation treatment short-course (DOTS) to ensure adherence. This study assessed the factors associated with interruption of treatment among patients on DOTS in Nandi County, Kenya.

**Methods:**

we reviewed medical records and interviewed randomly selected persons from the County TB register, among those initiated on TB treatment between 1st January 2013 and 30th June 2014. Data on socio-demographics, clinical characteristics, behavioral factors, family support, health system factors, income, and lifestyle and treatment interruption (i.e., therapy discontinuation ≥ 2 weeks) were collected. We calculated odds ratios (OR) and 95% confidence intervals (CI) to evaluate factors associated with TB interruption and performed multivariable logistic regression to examine independent risk factors.

**Results:**

from a total of 1,287 records in the TB register, we randomly selected 280 patients for interview, out of whom 252 were traced. Of the 252 participants interviewed, 149 (59.1%) were males and the mean age was 40.0 (SD ± 15.3) years. Seventy-eight (31.0%) interrupted treatment. Treatment interruption was associated with personal monthly income ≤ 10,000 Kenya shillings ($100) (AOR 4.3, CI = 2.13-8.62) compared to income > 10,000 Kenya shillings, daily alcohol consumption of > 3 days per week (AOR 3.3, CI = 1.72-6.23) compared to consumption of ≤ 3 days per week and average waiting time at the health facility ≥ 1 hour (AOR 3.5 CI = 1.86-6.78) compared to waiting time < 1 hour.

**Conclusion:**

we suggest expanding DOTS services to increase the number of service points for patients. This will probably reduce the waiting time by distributing the work load across many facilities. Intensifying patient counseling and education prior to initiation of treatment could also be adopted to cover effects of alcohol use during treatment and teach patients to take up some income generating activities.

## Introduction

Tuberculosis (TB) is a chronic infectious disease caused by various species of the Mycobacterium genus. If untreated, an infected patient can infect an average of 10-15 persons in a year [1, 2]. In 2013, two billion people in the world were infected with TB, representing about a third of the entire world population [3]. In the same year, nine million people contracted TB and 1.4 million died of it [4]. In 2012, over 95% of TB deaths occurred in low and middle-income countries [5] and it was among the top three causes of death for women aged 15 to 44 years [6]. Of the more than nine million new cases of active TB that occur worldwide annually, approximately 30% are in Africa [6 ,7].

In 2012, Kenya was ranked 15th among the 22 countries on the list of high-TB burden countries by the World Health Organization (WHO) [8], with a case notification rate of 440 cases per 100,000 persons [9]. In 2011, Nyanza, Rift Valley Province and Nairobi contributed 56% of the total TB burden in Kenya [10]. Since 2009, Kenya has adopted the WHO strategy for TB control using a 6-month treatment regimen [11].

Interruption of treatment (therapy discontinuation ≥ 2 weeks) has been a major obstacle to treatment adherence, and is an important challenge for TB control. Inability to complete the prescribed 6-month regimen is an important cause for treatment failure, relapse, acquired drug resistance and on-going transmission of infection. Over the years, there has been increasing emphasis on direct observation treatment short-course (DOTS) to improve adherence, wherein each dose of treatment is given under the observation of a health worker. The adoption of DOTS has given impressive results with higher treatment success being reported from developing and industrialized countries [12-14].

Treatment interruption is a precursor to defaulting (therapy discontinuation for ≥ 4 weeks); it thus gives insight of what happens to patients prior to defaulting. This also provides an opportunity for early intervention in the course of treatment. TB interruption rates from as low as 1% in good health systems to as high as 70% in worse performing areas have been found [13, 15]. Studies conducted in Delhi, India and Nairobi, Kenya, found the average time to interrupt was six (± 3) weeks after initiation of treatment [16, 17]. Others have assessed factors associated with TB treatment interruption [13,17,18]. Treatment interruption has been associated with long transportation time to treatment centre, being male, patients with low level of information about TB, poor quality of communication between patients and health workers, distance to treatment centres, cigarette smoking and inadequate knowledge of TB treatment duration among patients [15,18, 19].

There are limited published data on factors associated with TB treatment interruption in Kenya. While the national treatment success rate was 87% in 2014, Nandi county lags behind at 77% [20]. We conducted this study to describe the frequency of treatment interruption and identify factors influencing this interruption in Nandi County, Kenya. The findings of this study will be used by the county to improve TB treatment outcomes especially in the screening of patients diagnosed with TB to identify those at risk of interrupting treatment.

## Methods

### Study setting, design and population

We conducted a cross-sectional study among TB patients who had been initiated on TB treatment between 1st of January 2013 and 30th June 2014 in Nandi County. Nandi county (population, 752,965) is comprised of five administrative sub-counties: Nandi Central, Nandi North, Nandi South, Nandi East and Tinderet [21] and has 138 health facilities, 45 of which are TB treatment sites [22]. Agriculture is the main economic activity: arable farming, cash crops and livestock keeping.

### Sample size determination

The estimated prevalence of interruption documented in Kenya in 2009 was 19% [23]. Using Cochran’s 1977 formula [24], the minimum sample size was estimated to be 236, assuming a power of 80% and a precision of 5%, which was increased to 260 allowing for 10% non-response.

### Sampling procedure

A sampling frame was developed by listing all patients initiated on TB treatment in Nandi County between January 1, 2013 and June 30, 2014 from the County TB register. We generated 260 random numbers and patients matching these assigned numbers were selected for interview. Any refusals were replaced with the next consecutive number on the registry list. For refusals for which there were more than three consecutive replacements were taken, a new random number was generated.

### Eligibility and exclusion criteria

In this study, a TB patient was defined as any person who had been diagnosed with TB based on clinical, microscopic or X-ray examination. Eligible patients were those resided within Nandi County who initiated TB treatment between 1st January, 2013 and 30th June, 2014 and were aged ≥ 14 years. We included patients that were initiated on TB therapy regardless of lab confirmation (smear positive or negative), HIV status, whether initial treatment or re-treatment, or whether patient had defaulted on therapy. We excluded patients aged ≤ 13 because we believed that they would not provide objective opinions. We also excluded patients that transferred outside the county for further treatment, as well as those reported to have died during and after completion of treatment because they could not be interviewed. Treatment interruption was defined as failure to adhere to prescribed TB medication for a period of two consecutive weeks or more by persons who were already on TB treatment, regardless of whether they returned to therapy or DOTS.

### Ethical approval and considerations

A consent form explaining the rationale and benefits of the study was used to seek informed consent from potential participants. Participants between 14 years and 17 years of age assented to the study and consent was obtained from their guardians. Participation was voluntary and participants could withdraw from the study at any stage without being penalized. No study participant was identified by name in any report from the study. Permission to conduct the study was obtained from Nandi county health department and ethical clearance was obtained from Jaramogi Odinga Oginga Teaching and referral hospital (JOOTRH) ethical review board ***(ERC.2/VOL.1 (103)).***

### Data collection

We traced the selected subjects to their homes and interviewed those found using semi-structured questionnaires. These were administered by trained data collectors using questionnaires in English, Kiswahili or Kalenjin depending on the dialect the respondent was most comfortable with. Questionnaires were back translated and pretested in non-participating units before use. Data collectors were mainly TB ambassadors, who are community health workers (CHW) with roles that include community diagnosis of TB and referral of TB patients. Data were collected on socio-demographic characteristics, clinical characteristics, family support, nutritional status, use of herbal medication, side effects experienced during treatment, knowledge on TB transmission and prevention, health system factors (care giver’s attitude towards patients, distance to treatment center and average waiting time at the facility), medication history, and lifestyle and interruption status (determined by interview). We conducted a medical record review of selected patients to assess patient follow-up done during the time the patients were on treatment. A check-list of the requirements of the recommended Ministry of Health schedules of tests and TB reviews was completed by abstracting record information into an abstraction form. The abstraction form collected data on patients’ socio-demographics, registration date, DOT provider, patient type, diagnostic tests done, HIV status and testing for patients and their partners, other medications used during treatment and treatment outcome from the register. Abstracted data were linked to patient questionnaire data for analysis, using each clinic registration number as a unique identifier.

### Data analysis

Descriptive statistics were generated with frequencies and proportions used to summarize categorical data and means and medians were used for continuous variables. We calculated a prevalence odds ratio (OR) and 95% confidence intervals (CI) to examine factors associated with treatment interruption. Independent factors were assessed using logistic regression, in which factors with a p-value of ≤ 0.15 were entered into the multivariate model. Factors with p-value < 0.05 were considered significant.

## Results

### Socio-demographic characteristics

**Figure 1:**
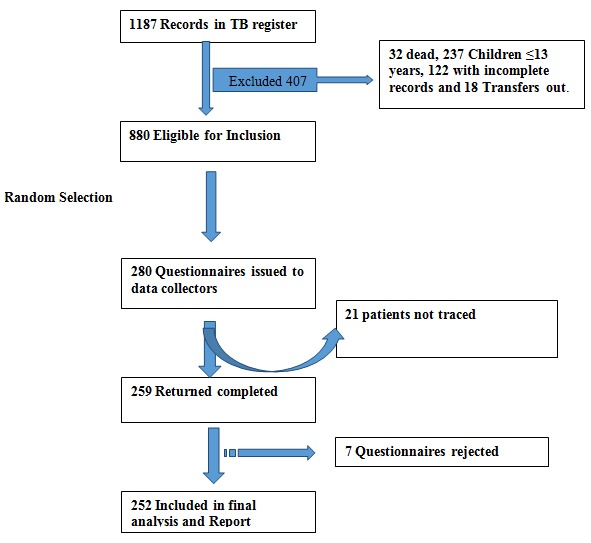
flow chart showing recruitment of patients into the study

There were 1,287 records from patients in the TB register initiated on treatment between January 1, 2013 and June 30, 2014 in Nandi County. From these, 407 patients were excluded: 193 patients were aged ≤ 13 years, 134 patients had incomplete records, 48 patients transferred out of the county, and 32 patients died prior to completion of treatment. Of the remaining 880, we randomly selected 280 patients to be interviewed. A total of 280 questionnaires were issued to data collectors. During data collection, five subjects declined interviews, three of whom indicated they did not have time for the interviews, the rest declined without giving reasons. None of the refusals was replaced more than three times. Of the questionnaires issued to data collectors, 259 were completed and 21werenot completed because the respondents were not traced. Seven questionnaires were rejected as a result of inconsistencies, not being completely filled, or were lacking unique identifiers. The remaining 252 were included, giving a response rate of 90% (252/280) (Figure 1).

Among the 252 respondents149 (59.1%) were male. The participants had a mean age of 40.0years (SD ± 5.3), with 69 patients (27.4%) aged 30-39 years. One hundred and fifty-two (60.3%) had at least primary level education. A hundred and sixty-one (63.9%) were self-employed, 64(25.4%) were unemployed while the remainder 27(10.7) were in formal employment. With respect to personal monthly income, 138(54.8) reported earning less than Ksh. 10, 000 ($100), 112(44.4%) between Ksh.10, 001 and Ksh 50,000, while only two (0.8) reported earnings above Ksh.50, 000. Nandi Central Sub County contributed the greatest proportion of patients (96 [36.5%]) while Tinderet Sub County contributed the fewest number of patients (15 [6.0%]).

Seventy-eight patients (31.0% [CI = 25.30-37.06]) reported to have interrupted treatment, compared to the remainder (69.0% [62.94-74.70]) who did not report interruption. Patients who interrupted treatment were similar to those who did not in terms of social demographic characteristics, except for those aged above 60 years (compared to age group 20-29) and those having a monthly income of Ksh.10,001-50,000 (compared to those earning ≤ Ksh.10,000). Having a personal monthly income of Ksh. 10,001-50,000 was protective against TB treatment interruption (OR = 0.20, CI 0.11-0.38), while being aged ≥ 60 years was associated with treatment interruption (OR = 3.10, CI 1.20-7.97) (Table 1).

**Table 1:** Socio-demographics of patients treated for TB in Nandi County, Kenya, 2013-2014

Characteristic	N (%)	With interruption (%)	Without interruption (%)	OR (95%CI)	*P* Value
**Total**	252 (100)	78 (31.0)	174 (69.1)		
**Sex**					
Female	103(40.9)	33(42.31)	70(40.23)	1.09(0.63-1.87)	0.864
Male	149 (59.1)	45 (57.7)	104 (59.8)	Ref	
**Age**					
Below 19	10 (4.0)	1 (1.3)	9 (5.2)	0.39(0.05-3.40)	0.397
20-29	59 (23.4)	13 (16.7)	46 (26.4)	Ref.	
30-39	69 (27.4)	26 (33.3)	43 (24.7)	2.14(0.97-4.69)	0.058
40-49	55 (21.8)	18 (23.1)	37 (21.3)	1.72(0.75-3.97)	0.202
50-59	29 (11.5)	6 (7.7)	23 (13.2)	0.92(0.31-2.74)	0.886
Over 60	30 (11.9)	14 (18.0.)	16 (9.2)	3.10(1.20-7.97)	0.019
**Marital status**					
Cohabiting	3 (1.2)	1 (1.3)	2 (1.2)	Ref.	
Married	143 (56.8)	46 (59.0)	97 (55.8)	0.95(0.08-10.73)	0.966
Other	20 (7.9)	8 (10.3)	12 (6.9)	1.33(0.10-17.28)	0.826
Single	86 (34.1)	23 (29.5)	63 (36.2)	0.73(0.06-8.44)	0.801
**Education level**					
Primary	152 (60.3)	53 (68.0)	99(57.0)	Ref.	
Secondary	72 (28.6)	19 (24.4)	53(30.5)	0.67(0.36-1.25)	0.206
Tertiary	28 (11.1)	6 (7.7)	22(12.6)	0.51(0.19-1.33)	0.170
**Occupation**					
Unemployed	64(25.4)	21 (26.9)	43 (24.7)	1.16(0.44-3.08)	0.933
Employed(Formal)	27(10.7)	8 (10.3)	19 (10.9)	Ref.	
Self employed	161(63.9)	49 (62.8)	112 (64.4)	1.04(0.43-2.53)	0.766
**Personal monthly income**					
0-10,000	138(54.8)	62(79.5)	76(43.7)	Ref.	
10,001-50,000	112(44.4)	16(20.5)	96(55.2)	0.20(0.11-0.38)	<0.001
Over 50,000	2(0.8)	0	2(1.1)	<0.0001	0.972
**Residence(District)**					
Nandi Central	92 (36.5)	23 (29.5)	69 (39.7)	Ref.	
Nandi East	41 (16.3)	13 (16.7)	28 (16.1)	1.56(0.68-3.59)	0.288
Nandi North	63 (25.0)	23 (29.5)	40 (23.0)	1.72(0.85-3.52)	0.125
Nandi South	41 (16.3)	15 (19.2)	26 (14.9)	1.80(0.80-4.04)	0.148
Tinderet	15 (6.0)	4 (5.1)	11 (6.3)	1.20(0.34-4.23)	0.775

### Patient diagnosis and follow-up

Most respondents (209 [82.9%]) had pulmonary TB and 217 (86.1%) of the respondents had DOTS supported by a family member. Diagnosis of TB was mostly through sputum examination, with 218 (86.5%) having had their sputum examination at diagnosis, 179 (71.0%) at three months and 166 (65.9%) at six months (end of follow-up). Sputum positivity declined from 67.4% at diagnosis to 1.8% at end of follow-up. One hundred and three patients (41.0%) had chest radiography. Almost all of the participants (244 [96.8%]) were tested for HIV, of whom 84 (34.4%) were HIV positive. Two hundred and thirty-one (91.7%) successfully completed treatment for TB, 17patients (6.8%) defaulted and four (1.6%) had treatment failures (Table 2).

**Table 2:** Ddiagnosis and follow-up for TB patients in Nandi County, 2013-2014

Variable	Total (%)	Patients with interruption (%)	Patients without interruption (%)
**Sputum results**			
**At diagnosis**			
Negative	71 (32.6)	18(30.0)	53(33.5)
Positive	147 (67.4)	42(70.0)	105(66.5)
**At 3 Months**			
Negative	165 (92.7)	45(93.8)	120(92.3)
Positive	13 (7.3)	3(6.2)	10(7.6)
**At the end**			
Negative	159 (98.1)	40(97.6)	119(98.3)
Positive	3 (1.9)	1(2.4)	2(1.7)
**Patient underwent X-ray examination**			
Yes	103 (41.0)	31 (39.7)	72 (41.6)
**HIV testing done**			
Yes	244 (96.8)	76 (97.4)	168 (96.6)
**HIV test results**			
Positive	84 (34.4)	34 (44.7)	50 (29.7)
**Patient on ART(HIV+)**			
No	1 (1.1)	0.0	1 (2.0)
Yes	83 (98.8)	35 (100)	48 (98.0)
**Partner tested (HIV)**			
No	173 (68.7)	51 (65.4)	122 (70.1)
Yes	79 (31.4)	27 (34.6)	52 (29.9)
**DOT provider**			
CHW	35(13.9)	13(16.7)	22 (12.7)
Family member	217(86.1)	65(83.3)	152 (87.4)
**TB type**			
Extra pulmonary TB	43 (17.1)	19 (24.4)	24 (13.8)
Pulmonary TB	209 (82.9)	59 (75.6)	150 (86.2)
**Distance from treatment site**			
≤10 Km	176	37(47.4)	139(79.9)
>10 Km	76	41(52.6)	35(20.1)
**Treatment outcome**			
Treatment complete and Cured	231 (91.7)	63(80.8)	178 (96.6)
Failure	4 (1.6)	2 (2.6)	2 (1.2)
Out of control	17 (6.8)	13 (16.7)	4 (2.3)

### Factors associated with TB treatment interruption in Nandi County, Kenya 2015

Patients who reported use of herbal medication during treatment (OR 2.6, CI = 1.36-5.00) were more likely to have treatment interruption compared to those who did not report use of herbs. Patients who reported experiencing side effects during treatment were more likely to have treatment interruption (OR 2.47, CI = 1.35–4.52) compared to those who did not report experiencing side effects. Other factors associated with increased odds of TB treatment interruption included having inadequate knowledge of TB transmission (OR 2.0, CI = 1.13-3.47) compared to having adequate knowledge on transmission; and average waiting time of ≥ 1hour at the treatment centre (OR 4.9, CI = 2.73-8.72) compared to average waiting time of ≤ 1 hour. Factors that decreased the odds of treatment interruption included being accompanied by a relative during visits to the treatment centre, (OR 0.49, CI = 0.29-0.85) compared to not being accompanied during visits; being informed of the diagnosis prior to initiation of therapy (OR = 0.3, CI = 0.11-0.76) compared to not being informed of the diagnosis and living ≤ 10 Km from treatment center (OR O.23, CI = 0.13-0.41 compared to living >10 Km from treatment center (Table 3). Using logistic regression to adjust for factors simultaneously, we found the following factors related to treatment interruption: personal monthly income ≤ 10,000 Kenya shillings ($100) (AOR 4.3, CI = 2.13-8.62) compared to personal monthly income > 10,000, frequent alcohol consumption of > 3 days in a week (AOR 3.3, CI = 1.72-6.23) compared to alcohol consumption of ≤ 3 days in a week, and long average waiting time at the treatment centre of ≥ 1 hour (AOR 3.5 CI = 1.86-6.78) compared to waiting time < 1 hour (Table 3).

**Table 3:** factors associated with TB treatment interruption in Nandi County, Kenya 2013-2014

Variable	Patients with interruption (%)	Patients without interruption (%)	POR (95%CI)	AOR (95%CI)
**Average reported waiting time at facility**			**4.9 (2.73-8.72)**	**3.6(1.86-6.81)**
≥1 Hour	54 (69.2)	55 (31.6)		
<1 Hour	24 (30.8)	119 (68.4)		
**Distance from treatment center**			**0.2 (0.13-0.41)**	
≤10 Km	37 (47.4)	139 (79.9)		
>10 Km	41 (52.6)	35 (20.1)		
**Age at initiation of treatment**			0.8 (0.48-1.40)	1.4(0.70-2.60)
<40 years	40 (41.3)	98 (56.3)		
≥40 years	38 (48.7)	76 (43.7)		
**Ever accompanied by Family member**			**0.5 (0.29-0.85)**	0.6(0.32-1.16)
Yes	33 (42.3)	104 (59.8)		
No	45(57.7)	70(40.2)		
**Ever experienced side effects**			**2.5 (1.35-4.52)**	1.44(0.71-2.94)
Yes	60 (76.9)	100 (57.5)		
No	18 (23.1)	74 (42.5)		
**History of prior TB Treatment**			1.4 (0.60-2.97)	
Yes	11 (14.1)	19 (10.9)		
No	67 (85.9)	155 (89.1)		
**Used herbal drugs during treatment**			**2.6 (1.36-5.01)**	
Yes	23 (29.5)	24 (13.8)		
No	55 (70.5)	150 (86.2)		
**Personal monthly income**			**5.0(2.67-9.34)**	**4.3(2.13-8.62)**
≤Ksh 10,000	62 (79.5)	76 (43.7)		
>Ksh 10,000	16 (20.5)	98 (56.3)		
**Alcohol use per week**			**5.0(2.83-8.92)**	**3.3(1.73-6.28)**
>3 days	51 (65.4)	47 (27.0)		
≤3 days	27 (34.6)	127 (73.0)		
**Sex**			1.1(0.63-1.87)	
Female	33 (42.3)	70 (40.2)		
Male	45 (57.7)	104 (59.8)		
**Highest Level of education**			0.6(0.36-1.10)	
Primary	25 (32.1)	75 (43.1)		
Post primary	53 (68.0)	99 (56.9)		

## Discussion

Our study found that a third of the TB patients in Nandi County had interrupted their TB treatment and factors associated with TB treatment interruption were alcohol use of more than 3 days in a week, personal monthly income ≤ 10,000 Kenya shillings ($100) and waiting time longer than 1 hour at treatment centre.

Interruption rates have been documented in other African countries, such as a study by Ibrahim et al, 2011 [18] that found one in every five patients in Plateau state in Nigeria had interrupted their TB treatment. In South Africa, Kandel et al, 2008 [15] found a TB treatment interruption rate of 47%. The high rate of interruption in our setting could be related to the high TB burden caused by HIV/AIDS pandemic [7, 17] against a low health workforce. This high number of patients interrupting treatment might have been because pre-treatment counseling was insufficient or of poor quality because health workers are overburdened. Insufficiency of counseling as a contributor to high interruption rates has also been advanced by Muture et al (Nairobi, Kenya, 2011) [17]. Inadequate pre-counselling could subsequently lead to poor patient practices that make patients vulnerable to failing to take their pills.

Behavioral factors, such as alcohol consumption, play an important role in determining interruption of TB treatment [4,14,18]. We found that frequent alcohol use increased the risk of interruption threefold. Individuals that take alcohol frequently could be drunk when they are required to take their next dose which can lead to interruption [25]. Alcohol also interferes with sleep pattern [26], impairs judgment and induces amnesia [27,28]. This might cause patients to forget to take their pills or forget appointments, resulting in treatment interruption. In 2005, Bagchi et al, in Mumbai found that alcohol consumption was associated with TB treatment interruption in those participants that were re-treatment cases [29]. Frequent consumption of large quantities of alcohol cause liver damage [30,31]. Concomitant use of anti-TB medication and alcohol worsens liver damage and can lead to treatment side effects [30]. Side effects will discourage patients from taking drugs, hence lead to treatment interruption. In our study we did not measure the volume of daily alcohol intake and the liver function tests were not documented in the records, therefore it was difficult to document any liver damage associated with alcohol use.

The socio-economic status of TB patients is also an important factor influencing TB treatment’s interruption. Our study found that in Nandi County, patients who reported to have a self-reported monthly income of ≤ 10,000 Kenya shillings ($100) were more likely to interrupt treatment. The average earning per employee in Kenya in 2016 was Ksh. 376,577.2 per annum (Ksh. 31,381.43 per month) [32]. This means a monthly income of Ksh.10, 000 is well below the average for the country. Similar findings have been reported by Dodor and Afenyandu, 2005 in Ghana [33]. In the study in Ghana, default from treatment was significantly associated with income per month, ability to afford supplementary drugs, availability of social support and problems relating with others while on treatment. In Kenya, the government supports TB treatment by purchasing drugs and providing free microscopy examinations, chest X-rays are paid for out of patients’ pockets. Patients also have other costs that they are responsible for, such as transport and opportunity cost during treatment from patients’ perspective. Hence, the decision to allocate money towards treating an illness makes these funds unavailable for food, clothing, housing and education. Persons with low incomes may first cater for basic needs before attending to an illness and this may lead to interruption of treatment.

We also found that patients who reported average waiting times of more than an hour at the treatment centre were more likely to interrupt treatment as compared to those who reported less than an hour. Similar findings are recorded in Tamatave, Madagascar by Comolet et al, 1998 [34], in which patients with a waiting time of > 1 hour were twice more likely to treatment interruption compared to patients with shorter waiting time. Long waiting time discourages clients from making subsequent return visits since it makes patients dissatisfied with services [35]. During the tracing of respondents, we observed that most patients lived more than 20 Km away from the treatment centers which further increases the time spent to access treatment since there is a poor road network and the means of transport are unreliable in most areas of Nandi county. Access is made worse during rainy seasons (April-May; October-November).

Our study findings should be interpreted in consideration of some limitations. Our study excluded patients treated for TB aged 13 years and below, therefore our findings are restricted to adults. We also excluded patients who died prior to completing treatment [32] because their inclusion would have necessitated proxies interviews which would influence the quality and reliability of the information gathered. There may have been interviewer and reporting bias, since the TB ambassadors (interviewers) are also involved in patient care. However, we sought to reduce this bias by intensive training of data collectors. Finally, as a cross-sectional study, our findings cannot be inferred to be causal, and further study over time on a cohort of patients would better identify risk of treatment interruption.

## Conclusion

We found that treatment interruption in Nandi County was associated with long waiting time at the treatment centre, frequent alcohol consumption and low income status of TB patients. We suggest that these factors be addressed to reduce treatment interruption by expanding DOTS services through creation of more TB treatment sites, intensify patient counseling and education prior to initiation of treatment and provision of financial information to encourage patients’ families to engage in income generating activities. Expansion of DOTS services could reduce the distance travelled to treatment centers, time used to travel, cost of travel and the waiting time at treatment facilities. This can be achieved by using other healthcare givers such as private clinics, chemists and nursing homes to implement private public mix. Patients should have education sessions on every visit not just one-off during therapy initiation, such sessions could address issues such as the dangers of alcohol use during treatment as well as encouraging their families to take up some income generating activities. Formation of patient support groups could also provide a good forum for sharing of experiences among patients as well as patient education by health workers.

### What is known about this topic

- Kenya is one of the countries with high incidence of TB;
- Treatment interruption is a precursor of defaulting in TB treatment.

### What this study adds

- This study provides an estimate of proportion of patients on TB treatment who interrupt treatment and also identifies the factors associated with TB treatment interruption in Nandi County, Kenya; it also recommends the possible ways of addressing the identified associated risk factors.

## Competing interests

The authors declare no competing interest.
